# Editorial: Horizons in neurorobotics 2022

**DOI:** 10.3389/fnbot.2024.1426355

**Published:** 2024-06-11

**Authors:** Florian Röhrbein, Hang Su, Poramate Manoonpong

**Affiliations:** ^1^Department of Computer Science, Chemnitz University of Technology, Chemnitz, Saxony, Germany; ^2^IBISC Laboratory, University of Evry Val Essonne, University of Paris-Saclay, Essonne, Evry, France; ^3^Embodied Artificial Intelligence and Neurorobotics Lab, SDU Biorobotics, The Mærsk Mc-Kinney Møller Institute, University of Southern Denmark, Odense, Denmark; ^4^Bio-Inspired Robotics and Neural Engineering Lab, School of Information Science and Technology, Vidyasirimedhi Institute of Science and Technology, Rayong, Thailand

**Keywords:** neurorobotic, human-robot interaction, robot intelligence, artificial intelligence, challenge

We are pleased to present the “*Horizons in neurorobotics 2022*” Research Topic of Frontiers in Neurorobotics. This Research Topic of articles represents a significant contribution to the field of neurorobotics, reflecting both the depth and breadth of research currently being undertaken in this area. The articles selected for publication in this Research Topic cover a wide range of topics, from fundamental theoretical developments to practical applications, each contributing uniquely to the advancement of neurorobotics.

This Research Topic features five articles from leading researchers, each of whom brings their unique perspective and expertise to the neurorobotics community. Below are brief summaries of the articles featured in this Research Topic:

- **When neuro-robots go wrong: a review** by Khan and Olds discusses the challenges and future directions of explainable AI within neuro-robotics, emphasizing the need for increased transparency in AI decisions within robotic systems. Robotic design is becoming increasingly influenced by biological brains, leading to the development of neurorobots. At the same time, there is a growing societal expectation for thorough explanations. In this article, the authors discuss the convergence of these two strands and their potential influence on the course of robotic design.
- **Inverse kinematics solution of 6-DOF manipulator based on multi-objective full-parameter optimization PSO algorithm** by Luo et al. introduces a novel Particle Swarm Optimization (PSO) algorithm for robotic manipulators through inverse kinematics solutions. This multi-objective, full-parameter optimization strategy notably enhances the precision and efficiency of the manipulator's movements. It incorporates multiple optimization objectives, such as minimizing position, posture, and joint angles, ensuring a comprehensive evaluation of possible solutions. Additionally, the study refines classical PSO features, including inertia weight, learning factors, and initialization strategies, which allow for more focused and effective navigation of the solution space to optimize the search process. Comparative testing against standard benchmarks and various modern algorithms demonstrates the proposed algorithm's superior performance, showing not only heightened accuracy in resolving inverse kinematics but also substantial gains in computational efficiency. Overall, this study contributes significantly to the fields of robotic motion control and optimization algorithms.
- **Deep causal learning for robotic intelligence** by Li proposes new architectures that integrate deep learning with causal inference (deep causal learning) to improve decision-making processes in robotic systems. The study envisions that deep causal learning offers a promising solution for overcoming challenges in real-world intelligent robots. This learning approach helps uncover causal relationships, enhancing our understanding of how data are generated. Such understanding boosts safety, reliability, and addresses ethical and societal concerns. Deep causal learning can be used to identify the key factors affecting a specific outcome, thereby simplifying domain-specific knowledge. It can be used to generate counterfactual predictions, enhancing decision-making and enabling complementary perception and understanding. Importantly, it organizes knowledge structures, facilitating stackable learning for improved perception, control, and scalability. The study also discusses causal cognition and statistical causal learning. Overall, it highlights a learning approach toward real-world robotic intelligence.
- **Continuous semi-autonomous prosthesis control using a depth sensor on the hand** by Castro and Dosen presents a new control system for myoelectric prostheses to improve the usability and dexterity for amputees. While conventional control approaches require the placement of surface electromyography (sEMG) and/or camera sensors on the user, this study employs a depth sensor placed on the dorsal side of the prosthetic hand, facilitating the semi-autonomous control of grasping actions. This practical approach eliminates the need for the user to wear additional equipment. The experimental results illustrate that the participants could use the system to grasp a variety of objects or object parts, approached from different directions, and placed either individually or within a cluttered environment. The system allows the participants to easily learn to grasp an object. Overall, the study represents an important step toward the development of a self-contained semi-autonomous system and reflects the advancement in the science and technology of embodied neurorobotics in clinical applications.
- **Recent advancements in multimodal human–robot interaction** by Su et al. reviews advances in human-robot interaction, focusing on multimodal approaches that enhance interactions between humans and robots. The authors thoroughly analyze the progress and upcoming trends in multi-modal HRI by examining possible I/O signals, modalities, and feedback mechanisms. Almost 100 research articles from all major application areas are summarized.

## Looking forward

The field of neurorobotics, which originally focused on embodied autonomous systems driven by brain-inspired mechanisms/models and/or actual biological systems, is evolving at an unprecedented pace, with new technologies and methodologies continually emerging. Looking forward, we are excited about the potential of these developments to further revolutionize our understanding and capabilities in this field. Given its multidisciplinary nature, integrating neuroscience, robotics, and artificial intelligence, the field can serve as a foundation for comprehending natural intelligence and translating it into nature-inspired machine intelligence.

## Conclusion

The articles in this Research Topic collectively push the boundaries of neurorobotics, offering new insights and tools that will undoubtedly influence future research directions. We hope that this Research Topic will serve as a valuable resource for both newcomers to the field and established researchers.

## Author contributions

FR: Writing – original draft, Writing – review & editing. HS: Writing – original draft, Writing – review & editing. PM: Writing – original draft, Writing – review & editing.

